# Hostile Takeover by *Plasmodium*: Reorganization of Parasite and Host Cell Membranes during Liver Stage Egress

**DOI:** 10.1371/journal.ppat.1002224

**Published:** 2011-09-01

**Authors:** Stefanie Graewe, Kathleen E. Rankin, Christine Lehmann, Christina Deschermeier, Leonie Hecht, Ulrike Froehlke, Rebecca R. Stanway, Volker Heussler

**Affiliations:** 1 Malaria Lab I, Department of Molecular Parasitology, Bernhard Nocht Institute for Tropical Medicine, Hamburg, Germany; 2 Institute of Cell Biology, University of Bern, Bern, Switzerland; University of Georgia, United States of America

## Abstract

The protozoan parasite *Plasmodium* is transmitted by female *Anopheles* mosquitoes and undergoes obligatory development within a parasitophorous vacuole in hepatocytes before it is released into the bloodstream. The transition to the blood stage was previously shown to involve the packaging of exoerythrocytic merozoites into membrane-surrounded vesicles, called merosomes, which are delivered directly into liver sinusoids. However, it was unclear whether the membrane of these merosomes was derived from the parasite membrane, the parasitophorous vacuole membrane or the host cell membrane. This knowledge is required to determine how phagocytes will be directed against merosomes. Here, we fluorescently label the candidate membranes and use live cell imaging to show that the merosome membrane derives from the host cell membrane. We also demonstrate that proteins in the host cell membrane are lost during merozoite liberation from the parasitophorous vacuole. Immediately after the breakdown of the parasitophorous vacuole membrane, the host cell mitochondria begin to degenerate and protein biosynthesis arrests. The intact host cell plasma membrane surrounding merosomes allows *Plasmodium* to mask itself from the host immune system and bypass the numerous Kupffer cells on its way into the bloodstream. This represents an effective strategy for evading host defenses before establishing a blood stage infection.

## Author Summary

Malaria is one of the most important infectious diseases in the developing world. It is caused by *Plasmodium* parasites, which are transmitted by female *Anopheles* mosquitoes during blood feeding. In the mammalian host, *Plasmodium* first develops within liver cells, growing from one parasite into many thousands. After this extensive replication, the parasites are released into the blood stream in vesicles termed merosomes that are surrounded by membrane. However, the origin of this membrane was unclear due to the absence of typical host cell membrane markers. Here, we analyzed several parasite- and host cell-derived membranes and show that the merosome membrane is of host cell origin. We also demonstrate that characteristic markers are lost from the host cell membrane once the parasite is liberated from its enclosure within the cell and moves freely in the host cell. The disappearance of membrane markers seems to be a consequence of the host cell death that is triggered toward the end of parasite development in the liver cell. The simultaneous induction of host cell death and retention of an intact host cell membrane enables the *Plasmodium* parasite to hide from the host immune system and thus to escape elimination before establishing a blood stage infection.

## Introduction

Despite considerable research and eradication efforts, malaria remains one of the most debilitating infectious diseases in the developing world. In 2008 alone, 247 million cases and nearly one million deaths were recorded [1]. Malaria is caused by *Plasmodium*, a protozoan parasite that is transmitted by infected female *Anopheles* mosquitoes. Transmission occurs during a blood meal and the parasite ultimately enters the bloodstream as a sporozoite. After traveling to the liver, it infects hepatocytes and replicates into several thousand merozoites. At the end of the liver stage, these merozoites are packaged into merosomes, which facilitate shuttling into the bloodstream [2]. Upon reaching the lung capillaries, the merosomes rupture and release their cargo of infectious merozoites [3] to infect erythrocytes. This mode of transition to the blood stage has been described previously [2] and while the general process of merosome formation is understood, many of its details are still unknown. One major point of controversy is the origin of the membrane surrounding the clusters of exoerythrocytic merozoites before and after merosome formation. In principle, there are three membranes from which it could stem: the parasite membrane (PM), the parasitophorous vacuole membrane (PVM) or the host cell membrane (HCM). It has been hypothesized that the merosome membrane derives from the host cell because this would be most advantageous to the parasite [2]. For one, it would not have to waste energy building an additional membrane that is only needed for a relatively short time. More importantly, being wrapped in HCM would camouflage the parasite as self, serving as a kind of Trojan horse as the merosomes enter the bloodstream. The vast majority of parasite antigens would be masked until the merozoites are released in the lung capillaries, and even then exposure time would be very brief since invasion of red blood cells is expected to occur quickly. However, attempts to prove that the HCM surrounds detached cells and merosomes by staining for typical hepatocyte surface markers were unsuccessful [3]. It has therefore been suggested that the membrane of merosomes derives from the PVM [4], [5]. Using live imaging we show that not the PVM but the HCM forms the membrane of detached cells and merosomes and that detachment of the infected hepatocyte begins almost immediately after the breakdown of the PVM. We also offer an explanation for the difficulty in detecting typical hepatocyte surface proteins in the merosome membrane: upon breakdown of the PVM, a transgenically expressed marker protein is quickly lost from the HCM. Protein biosynthesis appears to come to a halt, likely due to a lack of energy since we observe rapid disintegration of host cell mitochondria.

## Results

**Figure 1:**
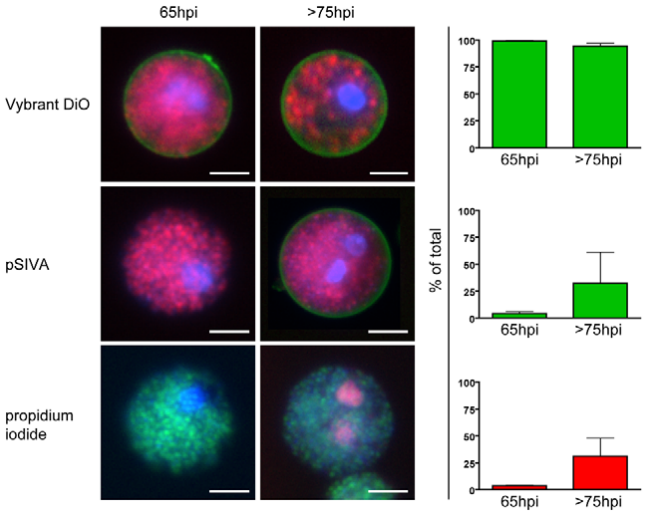
The membrane of freshly formed detached cells is intact and retains phosphatidylserine asymmetry. HepG2 cells were infected with fluorescent *P. berghei* parasites and detached cells were collected at either 65 or >75 hours of infection. They were then stained with the nuclear dye Hoechst 33342 (blue) and several additional live stains to characterize the nature of the surrounding membrane. The existence of a membrane in general was shown by staining with Vybrant DiO (green) in cells infected with *P. berghei*-mCherry (red, top panel). The presence of phosphatidylserine in the outer membrane leaflet, an indicator for ongoing cell death, was tested using pSIVA (green) in cells infected with *P. berghei*-mCherry (red, middle panel). Propidium iodide (red) is a membrane-impermeable nuclear stain and served to check the integrity of the membrane of *P. berghei*-GFP (green)-infected detached cells (lower panel). Quantification of the phenotypes (mean + SEM of three independent experiments) clearly shows that the membrane of freshly formed detached cells was intact and phosphatidylserine-negative while the membrane of older detached cells slowly lost phosphatidylserine asymmetry and integrity. Total n for 65hpi - Vybrant DiO: 712, pSIVA: 1483, PI: 1291. Total n for 75hpi - Vybrant DiO: 899, pSIVA: 874, PI: 1564. Bars  = 10 µm; WF (wide-field).

As a starting point, we examined the membrane surrounding detached cells and merosomes more closely. Since merosomes have been shown to directly bud from detached cells [2], [3], they share the same membrane and no distinction was made between the two. However, care was taken to ensure they indeed possessed the same membrane features. Initially, we infected HepG2 cells with *P. berghei*-mCherry or -GFP parasites and collected detached cells and merosomes at early and late time points after formation. Several live stains were then used to evaluate the nature of the membrane: Presence of a membrane was confirmed via the membrane stain Vybrant DiO for both freshly formed and aging detached cells and merosomes (Figure 1, top panel). The distribution of phosphatidylserine (PS) as a marker of apoptosis was checked using pSIVA, which binds to PS exposed in the outer membrane leaflet and can be used under growth media conditions [6]. pSIVA staining demonstrated that detached cells and merosomes were initially mostly PS-negative but lost PS asymmetry by 75 hours post infection (hpi), a timepoint when the intracellular parasites have mainly disintegrated [2] (Figure 1, middle panel). In addition, aging detached cells and merosomes increasingly lost membrane integrity as shown by staining with the membrane-impermeable nuclear stain propidium iodide (Figure 1, bottom panel).

### The parasite membrane becomes the merozoite membrane

**Figure 2:**
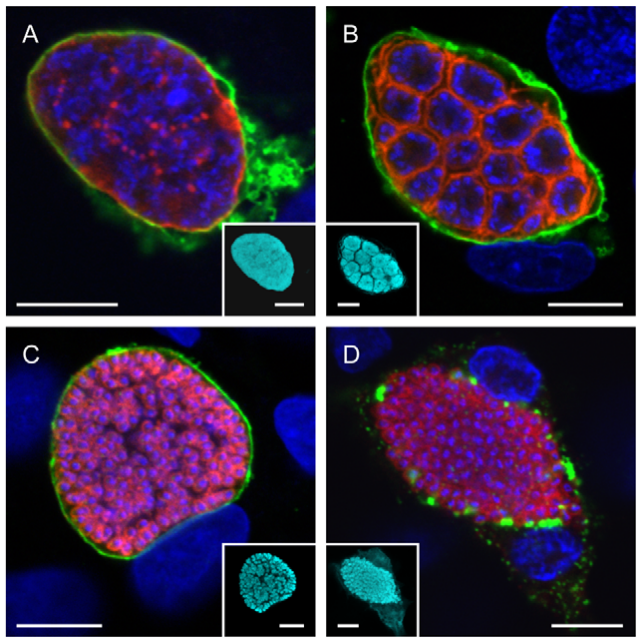
The parasite membrane becomes the merozoite membrane during the late liver phase. HepG2 cells were infected with *P. berghei* parasites and fixed at different time points after infection. They were then stained with an anti-MSP1 antibody to visualize the PM (red) and anti-Exp1 antibody to label the PVM (green). The parasite cytoplasm was labeled using a transgenically expressed protein (cyan) and the nuclei were stained with DAPI (blue). While the PM surrounded the parasite as a whole during the late schizont stage (A), it began to invaginate around groups of nuclei during cytomere formation (B). It eventually surrounded individual merozoites both before (C) and after (D) PVM breakdown. Representative images are shown. Bars  = 10 µm; CPS (confocal point scanning).

With the existence of a membrane verified, there were three candidate membranes from which it could originate: the PM, the PVM and the HCM. Moving from the inside out we first examined the fate of the PM during the late liver stage. HepG2 cells were infected with *P. berghei*-mCherry sporozoites and fixed at different time points after infection. They were then stained for immunofluorescence analysis with anti-MSP antibody to label the PM and anti-Exp1 antiserum to label the PVM. The parasite cytoplasm was visualized by staining with anti-RFP antibody and nuclei by staining with DAPI. Confocal images were taken and false-colored to facilitate assessment of colocalization: nuclei are shown in blue and the PVM in green while the PM is displayed in red and the parasite cytoplasm in cyan (Figure 2). In the schizont stage, both the PM and the PVM surround the syncytic parasite (Figure 2A). Shortly afterwards, the PM begins to invaginate around groups of nuclei to form the cytomere stage (Figure 2B). This invagination proceeds until the PM forms the membrane of the individual merozoites (Figure 2C). We consistently observed that only once this process was completed, did we see PVM rupture as could be determined by the loss of a clear Exp1-staining pattern (Figure 2D). This is the last step before cell detachment but no PM staining was detectable anywhere except around individual merozoites, discounting the PM as the membrane surrounding the detached cell and merosome. Even though MSP1 was exclusively detected in the merozoite membrane, it cannot be completely ruled out that the host cell membrane surrounding merosomes and detached cells could contain other parasite components through modification of the existing membrane. However, so far no proteins of parasite origin could be detected (data not shown). In any case, the important notion is that the membrane of merosomes and detached cells is not of parasite origin.

### The membrane of the parasitophorous vacuole disintegrates

**Figure 3:**
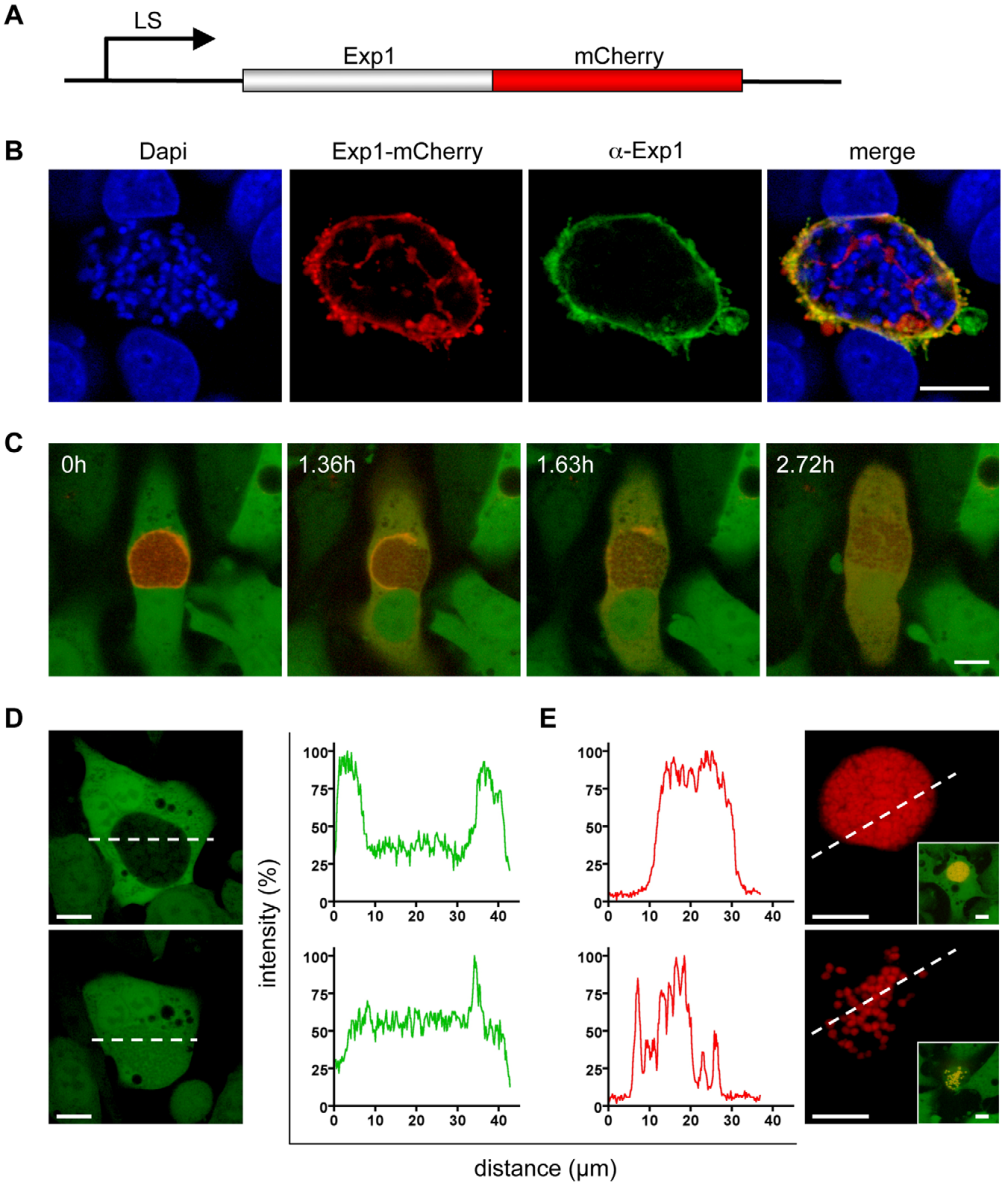
The membrane of the parasitophorous vacuole breaks down entirely. **A** The coding sequence of the Exp1 protein, which localizes to the PVM, was fused to the coding sequence of mCherry. Expression of the construct in the parasite was driven by a liver stage-specific promoter (LS). **B** Correct localization of the expressed fusion protein was confirmed by fixation and immunofluorescence staining of infected cells with anti-Exp1 antibody (green). Nuclei were stained with DAPI (blue) and the fusion protein with anti-RFP antibody (red). Bar  = 10 µm, CPS. **C** HepG2-GFP cells were infected with *P. berghei*-Exp1-mCherry parasites and imaged starting at 62 hpi. Stills from a representative movie (Video S1) clearly show that the PVM broke down completely. Quantification of this event confirmed that 100% of detached cells had cytosolic distribution of mCherry-Exp1. n = 20 from six independent experiments, bar  = 10 µm, CLS (confocal line scanning). **D** and **E** HepG2-GFP cells were infected with *P. berghei*-mCherry parasites. Representative cells were chosen to generate fluorescence profiles of both channels before and after PVM breakdown. Loss of the PVM barrier could be seen by entry of host cell GFP into the parasitophorous vacuole (D) and the spread of the densely packed merozoites into the host cell cytoplasm (E, see also Video S2). Bar  = 10 µm, CLS.

Next we investigated the fate of the PVM upon merozoite formation more closely. For live imaging we generated a transgenic *P. berghei* strain that expresses Exp1, a protein that localizes to the PVM, fused to mCherry (*P. berghei*-Exp1-mCherry, Figure 3A). Since constitutive expression of the fusion protein led to a developmental arrest of the parasite (RRS, unpublished observation), we used a liver stage-specific promoter for expression [7]. Correct localization of the fusion protein was confirmed by immunofluorescence analysis of HepG2 cells infected for 48 hours with *P. berghei*-Exp1-mCherry parasites. Infected cells were stained with anti-RFP (also detects mCherry) and anti-Exp1 antibodies and imaged confocally, allowing visualization of both endogenous and recombinant Exp1 (Figure 3B). Overlaying the confocal images shows almost complete colocalization, indicating that the *P. berghei*-Exp1-mCherry parasite line is a useful tool for imaging the PVM. We therefore infected GFP-expressing HepG2 cells with *P. berghei*-Exp1-mCherry sporozoites and observed parasite development in a confocal live setup starting at 62 hpi. Stills of a representative movie (Video S1) are shown in Figure 3C. In the first image of the series, merozoites have already formed and can be seen in negative (3C, 0h). They are surrounded by the clearly delineated PVM, but are released into the host cell cytoplasm as the PVM breaks down (3C, 1.36h). The PVM disintegrated further (3C, 1.63h) until all mCherry fluorescence was distributed equally throughout the host cell (3C, 2.72h). Detached cells and merosomes resulting from six independent *P. berghei*-Exp1-mCherry infections were examined and showed the same fluorescence pattern (n = 20). All cells were followed until detachment to ensure that the parasite contained within was viable and completed development. Additionally, detached cells and merosomes were collected from the supernatant of *P. berghei*-Exp1-mCherry-infected cell cultures that had not been imaged and examined for the presence of a PVM. In all cases, the PVM had disintegrated entirely (data not shown). The complete breakdown of the PVM before detachment was also obvious when looking at the GFP fluorescence profile across a line through the host cell. GFP distribution at an earlier time point was restricted to the cytoplasm of the host cell and was excluded from the PV by the PVM (Figure 3D, top panel). At a later time point, GFP was evenly distributed through both the host cell cytoplasm and the PV lumen, demonstrating that the PVM as a barrier had disappeared (Figure 3D, lower panel). A similar phenomenon could be seen in reverse when examining the fluorescence profile of *P. berghei*-mCherry, which carries mCherry in the parasite cytosol. Initially, fluorescence was confined to a peak corresponding to the densely packed area of merozoites within the PV (Figure 3E, upper panel). Later the fluorescence peaks were much more evenly distributed because merozoites had spread throughout the cell (Figure 3E, lower panel). They appeared to move more freely and much faster (Video S2). This rapid movement of merozoites also served as a marker of parasite viability, as injection of detached cells or merosomes containing motile merozoites into mice reproducibly led to blood stage infections [8]. In summary, since the PVM disintegrates entirely before and during detachment of the host cell, it also can be dismissed as the origin of the detached cell and merosome membrane.

### The host cell membrane forms the membrane of detached cells and merosomes

**Figure 4:**
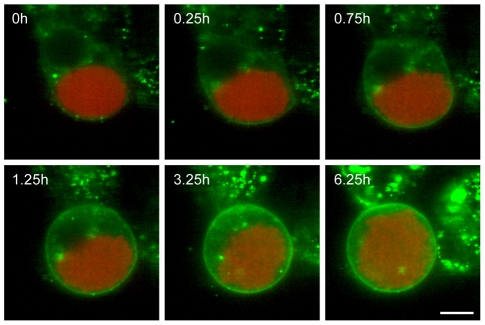
The host cell membrane forms the membrane of the detached cell. HepG2 cells were infected with *P. berghei*-mCherry (red), stained with Vybrant DiO (green) and imaged starting at 62 hpi. Stills from a representative movie (Video S3) are shown and demonstrate that the HCM became the membrane surrounding the detached cell. n = 34 (of which 10 covered development from PVM breakdown to complete detachment) from 11 independent experiments, bar  = 10 µm, CLS.

As we could show that neither the PM nor PVM are the source of the detached cell and merosome membrane, the HCM was the obvious remaining candidate, despite the fact that previous attempts to stain hepatocyte surface markers were unsuccessful [3]. Therefore we chose a different approach by labeling the HCM with the general membrane stain Vybrant DiO before PVM breakdown and detachment. We confirmed that Vybrant DiO indeed labeled the hepatocyte membrane by transfecting HepG2 cells with pDisplay-mCherry, which encodes mCherry fused to a secretion signal sequence and a transmembrane domain that anchors it in the membrane (Figure S1A). Co-staining of the transfected HepG2 cells with Vybrant DiO and subsequent confocal imaging showed that both the plasma membrane and intracellular membranes were clearly labeled (Figure S1B). The overlay image shows colocalization of the Display-mCherry protein and Vybrant DiO in the HCM, therefore verifying the suitability of Vybrant DiO for our experimental setup. Additionally, infection of HepG2 cells with *P. berghei*-Exp1-mCherry, which has a red-fluorescent PVM, followed by staining with Vybrant DiO showed that the dye does not label parasite membranes but is host cell-specific (Figure S1C). Live imaging of HepG2 cells infected with *P. berghei*-mCherry and stained with Vybrant DiO from 62 hpi allowed visualization of the membrane around the detaching cell (Figure 4 and Video S3). Since only host cell membrane material is stained, the membrane must be either the HCM or an additional internal membrane that is built specifically for this purpose. To exclude the presence of such a membrane, infected cells were examined by electron microscopy during the late liver stage (Figure S1D). While the HCM, PVM and PM are easily detectable, there was no evidence for any additional pre-existing or arising membrane. Therefore, the membrane of detached cells and merosomes must be derived from the HCM. This led us to test why this membrane does not stain positive for hepatocyte surface markers.

### A transgenic membrane marker is rapidly lost from the host cell membrane upon PVM breakdown

**Figure 5:**
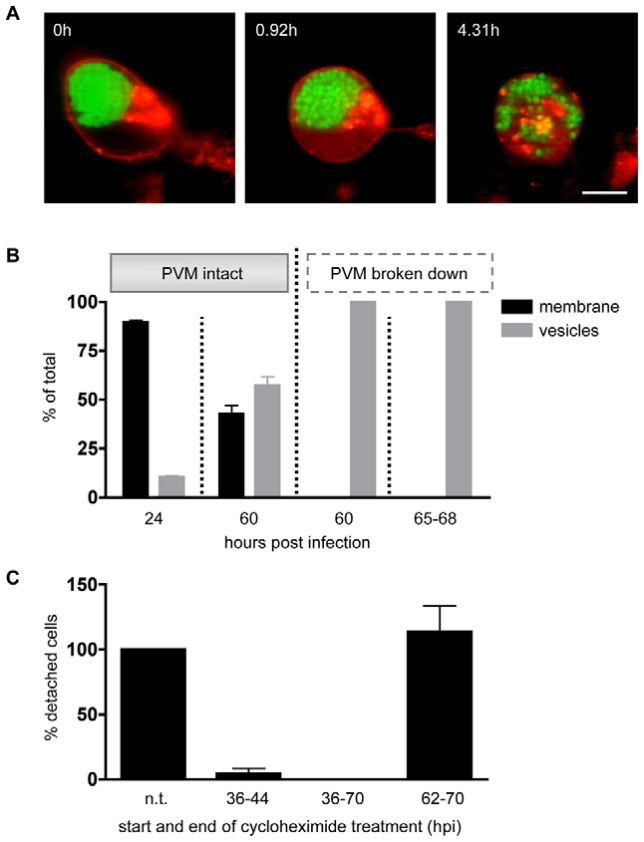
A host cell membrane protein is rapidly lost upon PVM breakdown. **A** HepG2 cells were transfected with pDisplay-mCherry and infected with *P. berghei*-GFP. From 62 hpi onwards cells were imaged. Stills from a representative movie (Video S4) show increasing loss of Display-mCherry from the HCM, eventually resulting in a spotty distribution throughout the cytoplasm of the detached cell. n = 3, bar  = 10 µm, CLS. **B** HepG2 cells were transfected with pDisplay-mCherry, infected with *P. berghei*-GFP and fixed at 24 and 60 hpi. Cells were stained for immunofluorescence analysis with anti-GFP and anti-RFP antibody. Late stages were additionally stained with anti-Exp1 antibody to determine the state of the PVM. Detached cells (65–68 hpi) were not fixed but collected from the culture supernatant and observed live. In all cases, the distribution of Display-mCherry was quantified (means of at least three independent experiments shown). Before PVM breakdown, Display-mCherry distribution was partly vesicular (grey columns) and partly membranous (black columns) while after PVM breakdown Display-mCherry could only be observed in vesicles. Total n for 24 hpi: 201, 60 hpi before PVM breakdown: 252, 60 hpi after PVM breakdown: 53, 65–68 hpi: 574. **C** HepG2 cells were infected with *P. berghei*-parasites and treated with 5 µg/ml cycloheximide for the indicated time periods. At 70 hpi detached cells were collected from the supernatant and counted (means of three independent experiments shown). The number of detached cells resulting from an untreated control was set to 100% and the other values were calculated in relation to it. Treatment at earlier stages blocked parasite development while treatment during the late stage had no effect on the number of detached cells in comparison to untreated samples. Viability of the host cells after cycloheximide treatment was confirmed by Live/Dead staining. Total number of detached cells: untreated: 2435, 36–44 hpi: 32, 36–70 hpi: 0, 62–70 hpi: 2714.

To address this issue, we transfected HepG2 cells with pDisplay-mCherry and then infected them with *P. berghei*-GFP sporozoites. Cells were then imaged in a confocal live setup beginning at 62 hpi. Stills of a representative movie (Video S4) show that the Display-mCherry protein initially localized to the HCM as expected (Figure 5A, 0h), but was quickly and progressively lost from the membrane upon PVM breakdown until it was distributed in patches throughout the cytoplasm of the detached cell (5A, 0.92h and 4.31h). For quantification, we compared infected cells before and around PVM breakdown. For this, we infected pDisplay-mCherry-transfected HepG2 cells with *P. berghei*-GFP sporozoites and fixed cells at 24 and 60 hpi. Samples were then stained for immunofluorescence analysis (IFA) with anti-RFP and anti-GFP antibodies. Cells that were fixed at 60hpi were additionally stained with an anti-Exp1 antiserum to determine if the PVM had already broken down. Because detached cells were floating they were collected from the supernatant of infected cell cultures at 65–68 hpi and examined directly. Localization of the Display-mCherry protein in infected cells was classified as either plasma membrane-bound (if the membrane was clearly stained) or entirely vesicular (Figure 5B). Early after transfection most cells showed plasma membrane localization of the Display-mCherry protein. Over time, the balance shifted towards a vesicular distribution, probably due to constant endocytosis processes in living cells. At the end of the liver stage, we still saw clear membrane localization of the Display-mCherry protein in 43% of the infected cells. However, once the PVM broke down, membrane localization of the fluorescent fusion protein was lost in all cells examined, both at 60 hpi and in detached cells and merosomes. This confirms the live imaging results that the Display-mCherry protein is lost from the HCM upon PVM disintegration. To test whether this phenomenon also occurs for proteins that are native to hepatocytes, we transfected HepG2 cells with an expression plasmid that encodes the human insulin receptor, which is known to localize to the hepatocyte plasma membrane [9], fused to GFP (IR-GFP) [10]. We confirmed the HCM localization by co-transfection with pDisplay-mCherry followed by confocal imaging (Figure S2A). Stills of a representative movie (Video S5) from time-lapse imaging of transfected, *P. berghei*-mCherry-infected HepG2 cells starting at 50 hpi showed that IR-GFP is found in the HCM during the schizont stage (Figure S2B, 0h) and early stages of merozoite formation (Figure S2B, 13.94h). However, upon breakdown of the PVM and detachment of the host cell, the distribution of IR-GFP becomes granular (Figure S2B, 16.55h) and it gradually disappears from the HCM. This was confirmed by fixing transfected, *P. berghei*-mCherry-infected cells at 48 hpi and as detached cells and staining them for immunofluorescence analysis with anti-GFP antibody (Figure S2C). While IR-GFP was present in the membrane of the host cell at the schizont stage (left panel), it was not found in the membrane of detached cells and merosomes (right panel). In addition, we also examined the fate of the major histocompatibility complex I (MHCI) protein. *P. berghei-*mCherry-infected cells were fixed at 48 hpi and as detached cells and stained for immunofluorescence analysis with anti-MHCI antibody (Figure S2D). Again, the membrane protein was detectable in the HCM at the parasite schizont stage (left panel), but was lost from the membrane of detached cells and merosomes (right panel). In conclusion, both transgenically expressed marker proteins and endogenous hepatocyte proteins disappear from the HCM upon PVM breakdown and detachment of the host cell.

We hypothesized that this might be due to an immediate arrest of protein biosynthesis upon PVM breakdown. To test this hypothesis, we treated *P. berghei*-infected HepG2 cells with cycloheximide, which blocks translational elongation and therefore protein biosynthesis in eukaryotes [11]. If protein biosynthesis in the host cell and the parasite indeed ceases towards the end of the liver stage, treatment with cycloheximide should have no effect on the development of the parasite and detached cells should form at a normal rate. Since most infected cells detach between 65 and 70 hpi, cells were treated between 62 and 70 hpi. As a control, infected cells were treated starting at an earlier time point (36–44 hpi or 36–70 hpi) when the parasite actively replicates and protein biosynthesis is expected to be crucial. At 70 hpi, detached cells from all treatment regimens were collected from the cell culture supernatant, counted and graphed as percentages of an untreated control (Figure 5C). As anticipated, both short- and long-term treatment at an earlier time point resulted in a marked decrease in the number of detached cells. In contrast, treatment during the late stage had no impact on detached cell formation. To ensure that the drop in detached cell formation after early-stage treatment was not due to host cell death, a live/dead-assay was performed on treated cells. No increase in host cell death was observed in relation to the untreated control (data not shown).

### Host cell mitochondria disintegrate after PVM breakdown

**Figure 6:**
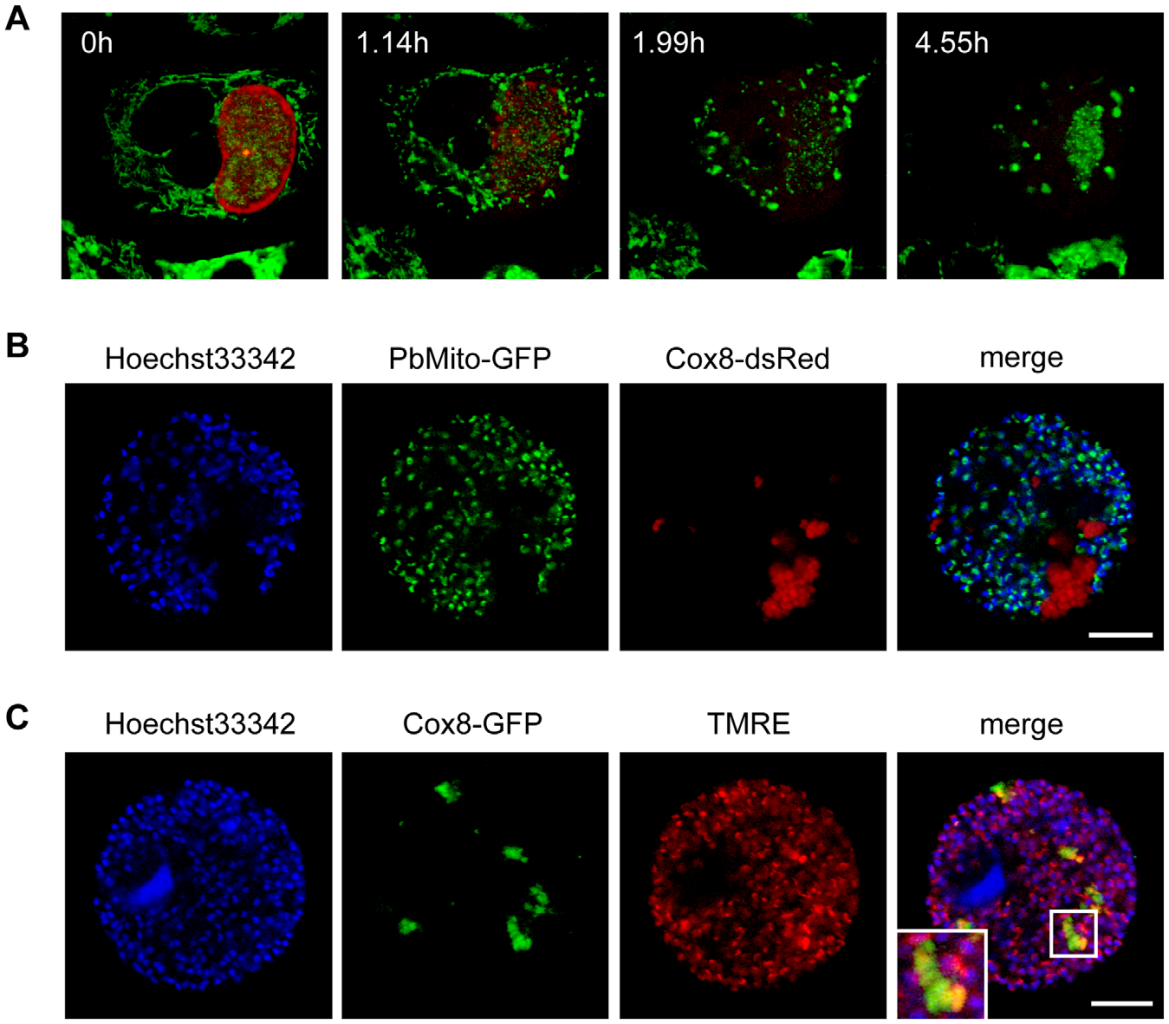
Host cell mitochondria disintegrate shortly after PVM breakdown. **A** HepG2 cells were infected with *P. berghei*-Exp1-mCherry and stained with MitoTracker GreenFM at 62 hpi. Stills from a representative movie (Video S6) show that host cell mitochondria drew together and disintegrated. n = 17 from three independent experiments, bar  = 10 µm, CLS. **B** pDsRed1-N1-Cox8-transfected HepG2 cells were infected with *Pb* ^c^GFP_MITO_ parasites to visualize parasite mitochondria (green). Detached cells were collected at 68 hpi and stained with Hoechst 33342 (blue). The overlay shows that the mitochondria remnants were of host cell origin. Representative images are shown. Bars  = 10 µm, CPS. **C** pEGFP-N1-Cox8-transfected HepG2 cells were infected with *P. berghei* parasites. Detached cells were collected at 68 hpi and stained with Hoechst 33342 (blue) and TMRE (red). Representative images show that most of the host cell mitochondria remnants (green) had no TMRE staining, indicating a loss of membrane potential. An enlarged part of the merged image (indicated by a white box) is shown in the inset. Bars  = 10 µm, CPS.

Protein biosynthesis can be inhibited during the normal course of apoptosis [12] and since host cell death during the late liver stage exhibits some features of apoptosis it is possible that this causes the observed loss of membrane proteins. However, since this mode of inhibition is usually dependent on caspase activation [12], which has been shown to be absent at this stage [13], other factors might play a role. We therefore examined if the apparent block in protein biosynthesis might be due to a lack of energy within the host cell. To test this, we infected HepG2 cells with *P. berghei*-Exp1-mCherry parasites and stained with MitoTracker GreenFM at 62 hpi to visualize mitochondria. Infected cells were then imaged in a confocal live setup. Stills of a representative movie (Video S6) are shown in Figure 6A. While the PVM was still intact, the host cell mitochondria displayed their typical morphology of a branched network (6A, 0h). Once the PVM broke down, host cell mitochondria appeared to draw together and began to form highly fluorescent clusters (6A, 1.14h). This process continued until only a few clusters were left in the detached cell (6A, 1.99h and 4.55h). To further characterize these mitochondrial clusters we labeled host cell mitochondria by transgenic expression of either GFP or dsRed. For this we used a plasmid construct carrying the respective fluorescent protein fused to the targeting sequence of the mitochondrial protein Cox8 (Figure S3A) [14]. Correct localization of the fluorescence markers was confirmed by co-staining with either MitoTracker GreenFM or tetramethylrhodamine ethyl ester (TMRE), a cell-permeant dye that stains active mitochondria (Figure S3B and 3C). To confirm that the mitochondrial remnants observed in detached cells are indeed of host cell and not of parasite origin, we infected Cox8-dsRed-transfected HepG2 cells with *Pb* ^c^GFP_MITO_ parasites, which express GFP fused to the targeting sequence of *Pb*Hsp60 [15]. Detached cells and merosomes were collected and confocal images taken (Figure 6B). The overlay image clearly demonstrates that the mitochondrial remnants are solely red-fluorescent and therefore of host cell origin. Functionality of the host cell mitochondrial remnants was then tested by infecting Cox8-GFP-transfected HepG2 cells with *P. berghei* parasites and staining detached cells and merosomes with TMRE (Figure 6C). It can be observed in the overlay image that in detached, infected cells very few host cell mitochondria were TMRE-positive, despite successful TMRE staining of parasite mitochondria. This indicates a massive loss of host cell mitochondria with intact membrane potential. This, in turn, means that most host cell mitochondria have lost their capability to produce ATP for intracellular processes like protein synthesis.

## Discussion

**Figure 7:**
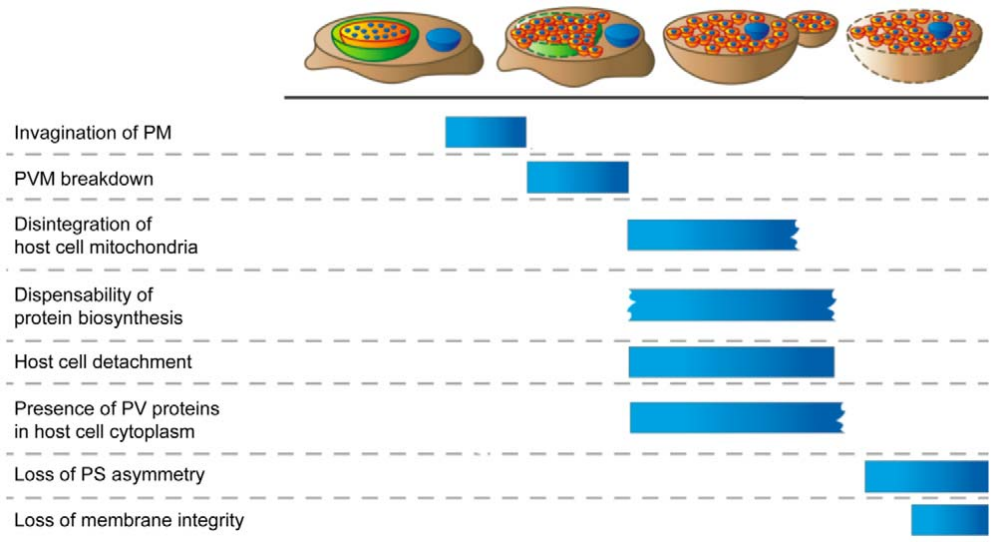
Sequence of events towards the end of the liver stage. After the PVM has invaginated around individual merozoites the PVM breaks down and releases the merozoites and the parasitophorous vacuole contents into the host cell cytoplasm. At this point host cell mitochondria begin to disintegrate, protein biosynthesis becomes dispensable and the host cell detaches. Merosomes bud from the detached cells, which initially retain both phosphatidylserine asymmetry and membrane integrity.

Although the liver stage of *Plasmodium* is an attractive target for both treatment and vaccination, we are still far from understanding even the basic biology of this parasite stage, partly because it is much more difficult to access than the blood stage. The existence of merosomes has previously been described as a transition mechanism from the liver stage to the blood stage [2], but many molecular details remained unknown. The possibility to efficiently generate transgenic parasite strains [16] has raised new possibilities to investigate this step in the parasite life cycle. It allowed us to identify the origin of the membrane surrounding detaching cells and merosomes by live imaging. The sequence of events uncovered towards the end of the liver stage is summarized in Figure 7. Initially, we characterized this particular membrane more closely using a series of live stains. The use of pSIVA, a newly developed annexin-based fluorescent biosensor [6], allowed us to stain for phosphatidylserine under culture conditions since it is polarity-sensitive and shows only low fluorescence when not bound to a membrane. We show that the membrane of freshly formed detached cells and merosomes remains intact and retains PS asymmetry. Upon aging of the detached cells and merosomes, the membrane slowly exposes PS and eventually loses integrity, confirming results from previous studies using *in vitro* and *ex vivo* merosomes [2], [3]. It is notable that the loss of membrane asymmetry and integrity occurs much faster in *P. yoelii ex vivo* than in *P. berghei in vitro* merosomes [2], [3]. This might be due to the obvious fundamental differences in the environment or the parasite strains.

### Neither parasite membrane nor PVM form the membrane of merosomes

Examination of the three potential parent membranes - PM, PVM and HCM - clearly ruled out the PM: through repeated invaginations during the late liver stage, it becomes the membrane of merozoites, confirming the suggestion from a previous publication [17]. Additionally, typical parasite membrane proteins were absent from the membrane of detached cells and merosomes, which therefore does not appear to be extensively rebuilt by the parasite.

Live imaging of a transgenic parasite strain whose PVM is visualized by expression of an Exp1-mCherry fusion protein then demonstrated that the PVM disintegrates entirely before detachment. It is therefore also not the membrane in question. This fits with the circumstantial evidence from previous publications [18]. Breakdown of the PVM was suggested as early as 25 years ago, after a mixture of host cell and parasite material had been observed within host cells by electron microscopy (EM). These observations have recently been confirmed and, in addition, host cell organelles have been observed in intravital merosomes [3]. Furthermore, the abundant PVM protein UIS4 could not be detected on merosomes [3], supporting our finding that the PVM is not the membrane surrounding detached cells and merosomes.

Live imaging always carries the risk of artifacts generated by phototoxicity due to prolonged exposure times. However, we excluded that the observed breakdown of the PVM was a consequence of photodamage for several reasons. First, merozoites in the resulting detached cells moved rapidly (compare Video S4). This rapid movement is a marker of parasite viability since injection of illuminated detached cells and merosomes containing motile merozoites into mice consistently results in blood stage infection [8]. Interestingly, the movement of merozoites appears to become far more pronounced once the PVM has ruptured, indicating that active movement was constrained by the PVM barrier. Second, a cytosolic distribution of Exp1-mCherry, indicating PVM breakdown, is observed also in detached cells and merosomes that have developed without exposure to laser light (data not shown). Finally, in our hands photodamage virtually always manifested as an arrest in development with the parasite exhibiting either a high level of vacuolization or undefined aggregates. Neither of these features are present in the parasites that successfully disrupt the PVM and enter the host cell cytoplasm. Also, it has been observed that merozoites that are finally liberated from merosomes are viable since they initially exclude propidium iodide and do not expose PS (VH, unpublished observations).

So far it is not known how breakdown of the PVM and egress of the parasite are triggered. The only protein known to be involved so far is the liver specific protein 1 (LISP1) of *P. berghei* [19]. It associates with the PVM and is necessary for its disruption, but the underlying mechanism remains unclear. Other likely mediators are proteases since PVM breakdown is blocked by the general protease inhibitor E64 [2]. In *T. gondii*, a perforin-like protein and changes in intracellular ion concentrations have been shown to orchestrate the disintegration of the PVM [20], [21], [22], and similar mechanisms might act in *Plasmodium*. Further insight could also be gained from the study of *Plasmodium* blood stage egress. Here the parasite is also surrounded by a PVM that is disrupted almost simultaneously with the HCM to liberate merozoites. This process likely involves a protease cascade [23], [24]. Interestingly, while in the blood stage the HCM disintegrates shortly after the PVM breakdown, our data show that in the liver stage the HCM stays intact for an extended period of time after the merozoites have been released into the host cell cytoplasm. A premature breakdown of the HCM would result in merozoite liberation into the liver tissue and the space of Disse but not into the bloodstream. It is therefore essential for exoerythrocytic merozoites to preserve the HCM until merosomes are formed and transported across the endothelium to a blood vessel.

### The host cell membrane surrounds detached cells and merosomes as a protective barrier

The molecular effects on the host cell upon PVM breakdown have been discussed previously [2], [3]. While the cell exhibits certain signs of apoptosis such as cytochrome c release and DNA condensation, important hallmarks such as caspase activation and the loss of phosphatidylserine asymmetry are absent [2], [13]. We demonstrate here that soon after PVM breakdown, the mitochondrial network of the host cell begins to disintegrate. The few remaining mitochondria clusters only partially retain a membrane potential. This stands in contrast to EM images of *in vivo* merosomes, which are reported to show well-preserved mitochondria [3]. Since the EM observations have not been quantified, it might be that the provided image coincidentally shows one of the rare cases where we also see preservation of mitochondrial membrane potential but it might also reflect a general difference between *in vivo* and *in vitro* merosomes or even *P. berghei* and *P. yoelii*. The mechanism underlying the mitochondrial degeneration is unclear, but might be connected to the host cell cytoskeleton since a similar mitochondrial fragmentation is seen after the interruption of vimentin intermediate filaments [25]. Both the morphology and loss of membrane potential closely resemble characteristics of mitochondria during apoptosis [26]. Mitochondria are often central to the induction of apoptosis [27] and it is possible that the breakdown of the PVM results in their exposure to apoptotic triggers. Parasitophorous vacuole-localized proteases are likely released and activated and might cause host cell death. Also, specialized parasite proteins could actively disrupt mitochondrial integrity as known for several viruses and bacteria [28], [29], [30]. Interestingly, the observed parasite-dependent host cell death seems similar to the initial stages of apoptosis. It is possible that the quick disruption of mitochondria leads to the rapid depletion of ATP within the host cell and that this results in an early arrest of the apoptotic program due to its dependence on energy. Therefore, features that arise early during apoptosis are present but the cell lacks the energy to execute the final steps such as DNA fragmentation and caspase activation.

A depletion of available energy in combination with inhibitory factors triggered during apoptosis [12] would also explain why we observe an arrest in protein biosynthesis around this time. This in turn might cause the change in distribution of the transgenically expressed membrane marker protein Display-mCherry and the hepatocyte membrane proteins IR-GFP and MHCI. After PVM breakdown, the proteins are quickly lost from the plasma membrane and are soon only present in some residual vesicles. It is possible that apoptotic membrane breakdown also plays a role in this process. Considering this, it is not surprising that it has been impossible to detect endogenous hepatocyte surface markers on the merosomal membrane [3].

Being wrapped in HCM is an elegant method of immune evasion by the parasite: to reach the lung capillaries, where merosomes rupture and release their cargo [3], they must pass through liver tissue and blood vessels which are lined with Kupffer cells. The HCM protects the traveling merozoites from being recognized as foreign and thus prevents phagocytosis and the initiation of an immune response. The loss of MHCI from the membrane of detached cells and merosomes is especially interesting in this regard as it will likely prevent the recognition by T_H_1 cells. Therefore, the parasite is shielded from the host immune system until it has reached the lung capillaries and thus conditions that are favorable for the invasion of erythrocytes.

Employing this Trojan horse strategy might also be advantageous to the parasite during subsequent reinfections: upon formation of merosomes *in vivo*, some parasite material remains in the liver [2], [3] and immune cells are known to be recruited to these sites [3], [13]. If the HCM simply ruptured to release the parasite, the remaining cell material would appear necrotic to immigrating phagocytes and could activate an inflammatory immune response [31], [32], [33]. Instead, though, it initially remains intact and only slowly begins to expose phosphatidylserine, whereupon it is recognized by phagocytes as apoptotic. Antigen that is taken up from apoptotic cells usually promotes tolerance and does not cause an immune response unless significant danger signals are present [34], [35], [36]. In combination with the suppressive effect of the parasite blood stages [37], [38], these mechanisms might limit the immune response against the liver stage and might aid subsequent infections. Therefore, *Plasmodium* might exploit the HCM both for short- and long-term protection from the host immune response.

In conclusion, we demonstrate here that after the invagination of the PM around merozoites, the PVM breaks down and the merozoites are released into the host cell cytoplasm. At the same time, parasite-dependent host cell death is initiated that alters the host cell profoundly, including the rapid disintegration of mitochondria. It is suggested that this leads to an arrest in protein biosynthesis and an aborted version of apoptosis. Ultimately, the host cell detaches and merosomes bud off, while the intact HCM serves as protection from the host cell immune system, thus representing the final step in the exploitation of the hepatocyte by the parasite to its own survival. Clearly, the parasite modifies the host cell extensively both before and after PVM breakdown and further research is needed to understand the full extent of these manipulations.

## Material and Methods

### Ethics statement

This study was carried out in strict accordance with the guidelines of the German Tierschutzgesetz (TierSchG; Animal Rights Laws). Mice were obtained from Charles River Laboratories. The protocol was approved by the Department of Veterinary Affairs of the Hamburg state authorities (Permit Number: FI 28/06). Blood feeding was performed under ketavet/rompun anesthesia, and all efforts were made to minimize suffering.

### Parasite strains

All parasite strains used in the present paper have a *P. berghei* ANKA background. The wild type strain does not express any fluorescent proteins. *P. berghei-*mCherry and -GFP express mCherry or GFP under the constitutive eef1aa promoter and show cytosolic localization of the respective fluorescent protein [39]. In *P. berghei*-Exp1-mCherry the PVM protein Exp1 [2], [13], [40] is fused to mCherry and expression is driven by the late liver stage-specific promoter of the gene PBANKA 100300 (PB103464.00.0) [7], [41]. In the *Pb* ^c^GFP_MITO_ parasite strain, GFP is targeted to mitochondria by fusion with the predicted mitochondrial targeting sequence of *P. berghei* heat shock protein 60 (Hsp60, PBANKA_121400); the sequence was amplified using the oligonucleotides ATATGGATCCATGCTATCTAGATTGTGTGGG and ATATGGATCCTATTACATTTCTTCCTTTGGGTC. Expression of the fusion protein in the parasite is controlled by the eef1aa promoter.

### Parasite transfection

Transgenic *P. berghei* strains were generated as described previously [16]. Briefly, expression plasmids were introduced into blood stage schizonts using electroporation and transfected schizonts were then injected intravenously into mice for replication and selected with pyrimethamine.

### Culture of HepG2 cells

HepG2 cells were purchased from the European cell culture collection and kept in Minimum Essential medium with Earle's salts supplemented with 10% FCS, 1% penicillin/streptomycin and 1% L-glutamine (all from PAA Laboratories, Austria). They were cultured at 37°C and 5% CO_2_ and split using Accutase (PAA Laboratories, Austria).

### Transfection of HepG2 cells

HepG2 cells were harvested by Accutase treatment and 2x10^6^ cells were pelleted by centrifugation at 160g. They were resuspended in Nucleofector V solution (Lonza, Cologne, Germany) and transfected with 3–5 µg plasmid DNA using program T-28 of the Nucleofector transfection device according to the manufacturer's instructions. The mCherry-pDisplay-plasmid was generated by inserting the coding sequence of mCherry into the pDisplay vector (Invitrogen, Darmstadt, Germany) and kindly provided by Isabelle Tardieux (Universite Paris Descartes, Paris, France). The pDsRed1-N1-Cox8 and pEGFP-N1-Cox8 plasmids contain the respective fluorescence protein fused to the targeting sequence of the mitochondrial protein Cox8. The hIR-GFP plasmid was purchased from addgene (Addgene plasmid 22286) and originally created by Rowena Ramos [10].

### HepG2 cell infection

8x10^4^ HepG2 or HepG2-GFP cells were seeded into either glass bottom dishes (WillCo Wells BV, The Netherlands) or on cover slips in 24-well plates. At 12–24 hours after seeding, *P. berghei* sporozoites were isolated from the salivary glands of infected *Anopheles stephensi* mosquitoes and added to the HepG2 cells. After an incubation period of 2 hours the sporozoite-containing medium was removed and fresh medium was added. At the indicated time points, infected cells were prepared for imaging. Detached cells and merosomes were collected from the supernatant of infected cell cultures as a mixture. Since merosomes have been shown to bud from detached cells [2], [3], they share the same membrane and were therefore examined as one.

### Cycloheximide treatment of infected HepG2 cells

HepG2 cells were seeded at 8x10^4^ cells per well into 24-well plates and infected with *P. berghei* sporozoites. They were then treated with 5 µg/ml cycloheximide (Sigma-Aldrich, Taufkirche, Germany) for the indicated time periods. The medium was changed twice daily and detached cells were removed and counted at 70 hpi.

### Live stains

*Hoechst 33342* was added at 1 µg/mL for 10 min at 37°C and 5% CO_2_. *MitoTracker Green* (Molecular Probes, Invitrogen, Darmstadt, Germany) was added to a concentration of 300 nM and incubated for 30 min at 37°C and 5% CO_2_. *Propidium iodide* (Sigma-Aldrich, Taufkirche, Germany) was added at a concentration of 3 µM for 15 min at room temperature. *pSIVA* (kindly provided by Yujin Kim, University of Southern California) [6] was added at 20 µg/ml and incubated for 1 h at 37°C and 5% CO_2_. *TMRE* (Molecular Probes, Invitrogen, Darmstadt, Germany) was added to a concentration of 25 nM and incubated for 30 min at 37°C and 5% CO_2_. *Vybrant DiO* (Molecular Probes, Invitrogen, Darmstadt, Germany) was added at 5 µl/ml for adherent and 10 µl/ml for already detached cells and incubated for 1 h at 37°C and 5% CO_2_. For all stainings of adherent cells, the staining medium was removed after the incubation period and fresh medium was added. For detached cells, the staining solution was diluted by adding an equal amount of fresh medium before imaging.

### Indirect immunofluorescent staining

8x10^4^ HepG2 cells were seeded on cover slips in 24-well plates. At appropriate time points after infection cells were fixed in 4% paraformaldehyde/PBS for 20 minutes at room temperature. They were washed three times with PBS and subsequently incubated in ice-cold methanol for 10 minutes at −20°C. After washing with PBS, unspecific binding sites were blocked by incubation in 10% FCS/PBS for 1 h at room temperature. Primary antibodies were diluted in 10% FCS/PBS and incubated for 2 hours at room temperature. Cells were then washed three times with PBS and secondary antibodies in 10% FCS/PBS were added for 1 hour at room temperature. Cells were washed again with PBS and mounted on microscope slides with Dako Fluorescent Mounting Medium (Dako, Cambridgeshire, UK). For the staining of detached cells and merosomes, the cell culture supernatant was collected at 68 hpi and centrifuged at 160g for 5 minutes. The cells were then resuspended in 4% PFA/PBS and incubated for 20 minutes at room temperature. Afterwards, they were spun down at 160g for 5 minutes and resuspended in a small amount of 4% PFA/PBS. The solution was subsequently spread onto a microscope slide using a cytospin centrifuge at 800g for 4 minutes. The detached cells and merosomes were incubated with 4% PFA/PBS for 15 minutes and then washed three times with PBS. They were then incubated with ice-cold methanol for 5 minutes at room temperature. After washing with PBS, they were blocked and stained immediately as described above. For anti-MHCI staining, cells were incubated with anti-MHCI antibody in culture medium on ice for 30 minutes. They were then washed three times with culture medium and fixed in 4% paraformaldehyde/PBS for 10 minutes at room temperature. After washing with PBS, they were incubated in 0.25% Triton X/PBS for 10 minutes at room temperature. They were again washed three times with PBS and subsequently blocked and stained as described above. The following antibodies were used: mouse FITC anti-MHCI antibody (BD Biosciences, Heidelberg, Germany), rat anti-RFP antibody (Chromotek, Planegg-Martinsried, Germany), chicken anti-Exp1 antibody (Heussler Lab, BNI, Hamburg, Germany), mouse anti-MSP1 antibody (kindly provided by Anthony Holder), anti-rabbit AlexaFluor594 antibody (Invitrogen, Darmstadt, Germany), anti-mouse AlexaFluor488 antibody (Invitrogen, Darmstadt, Germany), anti-rat AlexaFluor594 antibody (Invitrogen, Darmstadt, Germany), anti-chicken Cy2 antibody (Dianova, Hamburg, Germany) and anti-mouse Cy5 antibody (Dianova, Hamburg, Germany). Nuclei were stained with 1 µg/ml DAPI (Invitrogen, Darmstadt, Germany) during the secondary antibody incubation period. All images of fixed cells were acquired using a confocal laser point scanning microscope (see below for specifications).

### Time lapse imaging

8x10^4^ HepG2 or HepG2-GFP cells were seeded into glass bottom dishes and infected with *P. berghei* sporozoites. At 60–62 hpi they were either imaged directly or stained as indicated. All time lapse imaging was performed using a confocal laser line scanning microscope (see below for specifications). Images were acquired every 10–20 minutes.

### Electron microscopy

HepG2 cells were infected with *P. berghei*-GFP parasites. They were subsequently FACS-sorted to enrich for infected cells and seeded on Thermanox cover slips. At 48 hpi cells were first washed twice with PBS and then fixed with 2% glutaraldehyde in sodium-cacodylate buffer pH 7.2. This was followed by a postfixation step with 1% osmium tetroxide and dehydration at increasing ethanol concentrations and propylene oxide. Cells were then embedded in an epoxy resin (Epon). Ultrathin sections were made with an Ultra Cut E microtome (Reichert Microscope Services, Depew, USA) and stained for imaging with uranyl acetate and lead citrate. Images were then acquired using a Tecnai Spirit transmission electron microscope (FEI, Eindhoven, The Netherlands) at an acceleration voltage of 80 kV.

### Immunofluorescence microscope specifications

For *wide-field* (WF) imaging, an Axiovert 200 base was used in combination with an X-Cite 120 Fluorescence Illumination System (EXFO, Mississauga, Canada). Excitation and emission light was passed through Chroma filter sets (AHF Analysentechnik AG, Tuebingen, Germany). Images were taken using a FAST1394 QICam camera (QImaging, Surrey, Canada) and the Openlab software. For all images, a Zeiss 20x LD A-Plan objective was used. For *confocal point scanning* (CPS) microscopy, an Olympus IX81 microscope was used. Images were acquired using an Olympus 100x UPlanSApo 1.4 Oil objective and the Olympus Fluoview software version 1.7b. Fluorescence was excited by a 405-nm diode laser (Olympus, Germany), a 488-nm argon laser (Olympus, Germany), a 559-nm diode laser (NTT Electronics, USA) and a 635-nm diode laser (Olympus, Germany). Emission light passed through a 405/Mar/5437635 filter set before detection via PMTs. *Confocal line scanning* (CLS) was performed with a Zeiss Observer. Z1 inverted microscope integrated into an LSM5 Live imaging setup. Images were acquired using a Zeiss 63x Plan-Apochromat 1.4 Oil objective and the Zeiss Efficient Navigation 2008 and 2009 software. Fluorescence was excited by a Sapphire 488-nm diode and a Compass 561-nm diode pumped laser (both Coherent, USA) and emission light passed through a 505-nm and a 575-nm long-pass filter before detection via a CCD line detector. During imaging, cells were kept in a CO_2_ incubator at 37°C.

### Image processing and analysis

After acquisition, contrast and brightness levels were optimized using either the Zeiss Efficient Navigation 2009 software or Adobe Photoshop CS 8.0. Images were only enhanced as a whole. Fluorescence profiles were generated using the Zeiss Efficient Navigation 2009 software.

### Accession numbers

Cytochrome c oxidase subunit VIII (Cox8), Protein/Entrez ID CAG28615; exported protein 1 (Exp1), PlasmoDB ID PBANKA_092670; green fluorescent protein (GFP), Protein/Entrez ID ADN93293; insulin receptor (IR), GenBank/Entrez ID M10051; mCherry, Protein/Entrez ID ACY24904; merozoite surface protein 1 (MSP1), Protein/Entrez ID AAA66185.

## Supporting Information

Figure S1 **Vybrant DiO localizes to the host cell membrane but does not stain parasite membranes. A** mCherry was inserted into the pDisplay vector, fusing it to a signal sequence targeting it for secretion and a transmembrane domain anchoring it in the host cell plasma membrane. Expression of the fusion protein was driven by the CMV promoter. **B** Labeling of the HCM by the general membrane stain Vybrant DiO was confirmed by transfection of HepG2 cells with pDisplay-mCherry before staining. Both the Display-mCherry protein and Vybrant localized to the HCM. Bar  = 10 µm, CPS. **C** HepG2 cells were infected with *P. berghei*-Exp1-mCherry parasites and stained with Vybrant DiO at 48 hpi. While the HCM was labeled by Vybrant, neither the PVM nor the PM were stained. Bar  = 10 µm, CPS. **D** HepG2 cells were infected with *P. berghei* parasites and fixed for electron microscopy at 48 hpi. The two magnifications show the HCM (grey arrows), the PVM (black arrows) and the PM (white arrows) but do not indicate the presence or formation of an additional membrane. Bar  = 5 µm for left image, bars  = 500 nm for magnifications.
(TIF)

Figure S2 **Endogenous host cell membrane proteins are lost upon formation of detached cells and merosomes. A** Localization of a GFP-tagged version of the human insulin receptor to the HCM was confirmed by co-transfecting HepG2 cells with pDisplay-mCherry and phIR-GFP. **B** HepG2 cells were transfected with phIR-GFP and infected with *P. berghei*-mCherry parasites. From 50 hpi onwards cells were imaged (Video S5). Stills show that during the schizont and early merozoite stage of the parasite (bright white signal and red signal in the insets), hIR-GFP (weaker white signal) localized to the HCM. Upon PVM breakdown and detachment of the host cell, though, hIR-GFP was lost from the membrane. The laser intensity that was needed to image the comparatively weak GFP signal led to a side-peak excitation of mCherry that therefore also appears in the green channel image. Bar  = 10 µm, CLS. **C** HepG2 cells were transfected with phIR-GFP and infected with *P. berghei*-mCherry parasites. Cells were fixed at 48 (left panel) and 68 (right panel) hpi and stained for immunofluorescence analysis with anti-RFP antibody to label the parasite cytoplasm (red) and anti-GFP antibody to label hIR-GFP (green). Nuclei were stained with Dapi (blue). While hIR-GFP was present in the HCM during the schizont stage, it did not localize to the membrane of detached cells (99%, n = 206). Bars  = 10 µm, CPS. **D** HepG2 cells were infected with *P. berghei*-mCherry parasites and stained live with anti-MHCI antibody at 48 (left panel) and 68 (right panel) hpi. They were then fixed and stained for immunofluorescence analysis with anti-RFP antibody to visualize the parasite cytoplasm (red) and DAPI to visualize nuclei (blue). MHCI was present in the HCM of infected cells during the parasite schizont stage but not in the membrane of detached cells (100%, n = 127). Bars  = 10 µm, CPS.
(TIF)

Figure S3 **Cox8-GFP and Cox8-dsRed localize to the host cell mitochondria. A** DsRed or GFP were fused to the targeting sequence of the mitochondrial protein Cox8. Expression in the host cell was driven by the CMV promoter. **B** HepG2 cells were transfected with pDsRed1-N1-Cox8 to label host cell mitochondria with dsRed (red). Cells were then stained with MitoTracker GreenFM (green) to confirm localization to mitochondria. Nuclei were labeled blue with Hoechst 33342. Bar  = 10 µm, CPS. **B** HepG2 cells were transfected with pEGFP-N1-Cox8 to label host cell mitochondria with GFP (green). Cells were then stained with TMRE (red) to confirm localization to mitochondria and Hoechst 33342 (blue) to visualize nuclei. Bar  = 10 µm, CPS.
(TIF)

Video S1 **The membrane of the parasitophorous vacuole breaks down entirely.** HepG2-GFP cells were infected with *P. berghei*-Exp1-mCherry parasites and imaged confocally starting at 62 hpi. The representative movie clearly shows that the PVM broke down completely. Bar  = 10 µm, CLS.
(AVI)

Video S2 **After PVM breakdown, merozoites rapidly move in the host cell cytoplasm.** HepG2-GFP cells were infected with *P. berghei*-mCherry parasites and imaged confocally starting at 62 hpi. After the breakdown of the PVM, the previously densely packed merozoites spread into the host cell cytoplasm. Bar  = 10 µm, CLS.
(AVI)

Video S3 **The host cell membrane forms the membrane of the detached cell.** HepG2 cells were infected with *P. berghei*-mCherry and imaged starting at 62 hpi. The representative movie demonstrates that the HCM became the membrane surrounding the detached cell. Bar  = 10 µm, CLS.
(AVI)

Video S4 **A host cell membrane protein is rapidly lost upon PVM breakdown.** HepG2 cells were transfected with pDisplay-mCherry and infected with *P. berghei*-GFP. From 62 hpi onwards cells were imaged. The representative movie shows increasing loss of Display-mCherry from the HCM, eventually resulting in a spotty distribution throughout the cytoplasm of the detached cell. Bar  = 10 µm, CLS.
(AVI)

Video S5 **IR-GFP is lost from the host cell membrane towards the end of the liver stage.** HepG2 cells were transfected with hIR-GFP and infected with *P. berghei*-mCherry. They were imaged confocally starting at 50 hpi. The movie demonstrates the loss of a continuous membrane localization of IR-GFP, resulting in a granular distribution upon detachment of the host cell. Bar  = 10 µm, CLS.
(AVI)

Video S6 **Host cell mitochondria disintegrate shortly after PVM breakdown.** HepG2 cells were infected with *P. berghei*-Exp1-mCherry, stained with MitoTracker GreenFM at 62 hpi and imaged confocally. The representative movie shows that host cell mitochondria drew together and disintegrated. Bar  = 10 µm, CLS.
(AVI)
