# Supplementary figures and images for: Hostile Takeover by Plasmodium: Reorganization of Parasite and Host Cell Membranes during Liver Stage Egress
Source: PLoS Pathog. 2011 Sep 1;7(9):e1002224. doi: 10.1371/journal.ppat.1002224 (PMC3164640; doi:10.1371/journal.ppat.1002224)

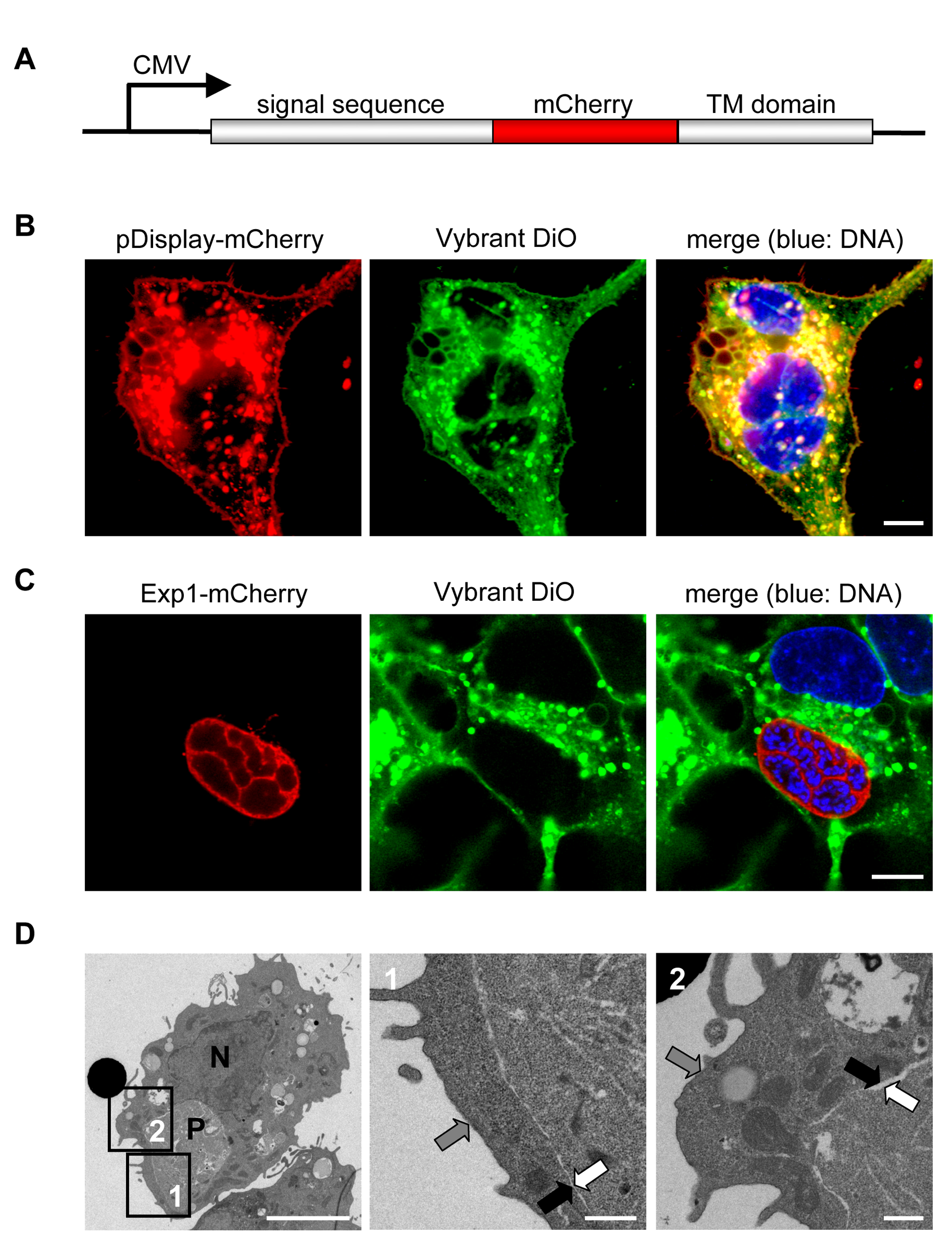

Supplement: Figure S1 — Vybrant DiO localizes to the host cell membrane but does not stain parasite membranes. A mCherry was inserted into the pDisplay vector, fusing it to a signal sequence targeting it for secretion and a transmembrane domain anchoring it in the host cell plasma membrane. Expression of the fusion protein was driven by the CMV promoter. B Labeling of the HCM by the general membrane stain Vybrant DiO was confirmed by transfection of HepG2 cells with pDisplay-mCherry before staining. Both the Display-mCherry protein and Vybrant localized to the HCM. Bar = 10 µm, CPS. C HepG2 cells were infected with P. berghei-Exp1-mCherry parasites and stained with Vybrant DiO at 48 hpi. While the HCM was labeled by Vybrant, neither the PVM nor the PM were stained. Bar = 10 µm, CPS. D HepG2 cells were infected with P. berghei parasites and fixed for electron microscopy at 48 hpi. The two magnifications show the HCM (grey arrows), the PVM (black arrows) and the PM (white arrows) but do not indicate the presence or formation of an additional membrane. Bar = 5 µm for left image, bars = 500 nm for magnifications. (TIF) [file ppat.1002224.s001.tif]

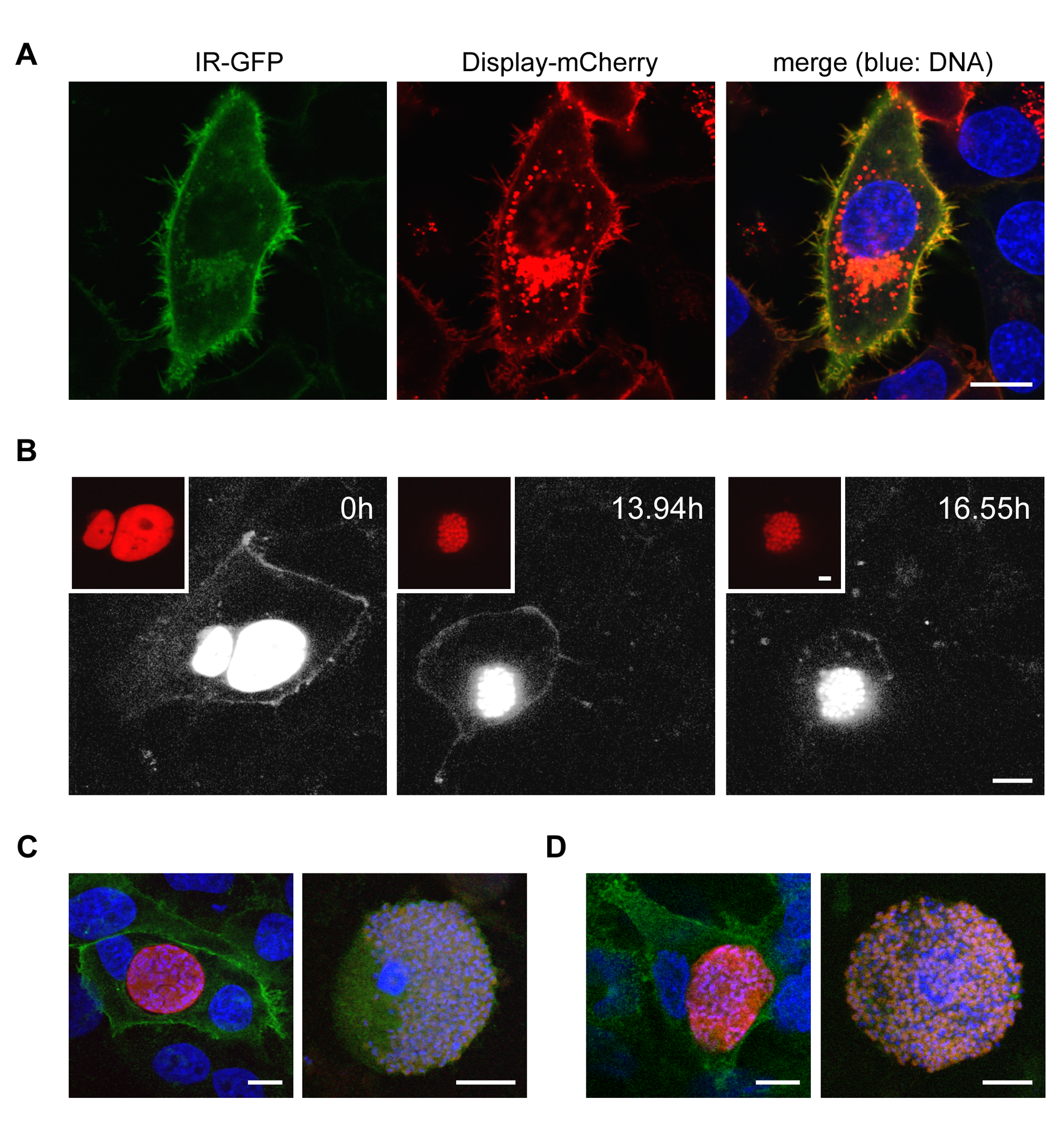

Supplement: Figure S2 — Endogenous host cell membrane proteins are lost upon formation of detached cells and merosomes. A Localization of a GFP-tagged version of the human insulin receptor to the HCM was confirmed by co-transfecting HepG2 cells with pDisplay-mCherry and phIR-GFP. B HepG2 cells were transfected with phIR-GFP and infected with P. berghei-mCherry parasites. From 50 hpi onwards cells were imaged (Video S5). Stills show that during the schizont and early merozoite stage of the parasite (bright white signal and red signal in the insets), hIR-GFP (weaker white signal) localized to the HCM. Upon PVM breakdown and detachment of the host cell, though, hIR-GFP was lost from the membrane. The laser intensity that was needed to image the comparatively weak GFP signal led to a side-peak excitation of mCherry that therefore also appears in the green channel image. Bar = 10 µm, CLS. C HepG2 cells were transfected with phIR-GFP and infected with P. berghei-mCherry parasites. Cells were fixed at 48 (left panel) and 68 (right panel) hpi and stained for immunofluorescence analysis with anti-RFP antibody to label the parasite cytoplasm (red) and anti-GFP antibody to label hIR-GFP (green). Nuclei were stained with Dapi (blue). While hIR-GFP was present in the HCM during the schizont stage, it did not localize to the membrane of detached cells (99%, n = 206). Bars = 10 µm, CPS. D HepG2 cells were infected with P. berghei-mCherry parasites and stained live with anti-MHCI antibody at 48 (left panel) and 68 (right panel) hpi. They were then fixed and stained for immunofluorescence analysis with anti-RFP antibody to visualize the parasite cytoplasm (red) and DAPI to visualize nuclei (blue). MHCI was present in the HCM of infected cells during the parasite schizont stage but not in the membrane of detached cells (100%, n = 127). Bars = 10 µm, CPS. (TIF) [file ppat.1002224.s002.tif]

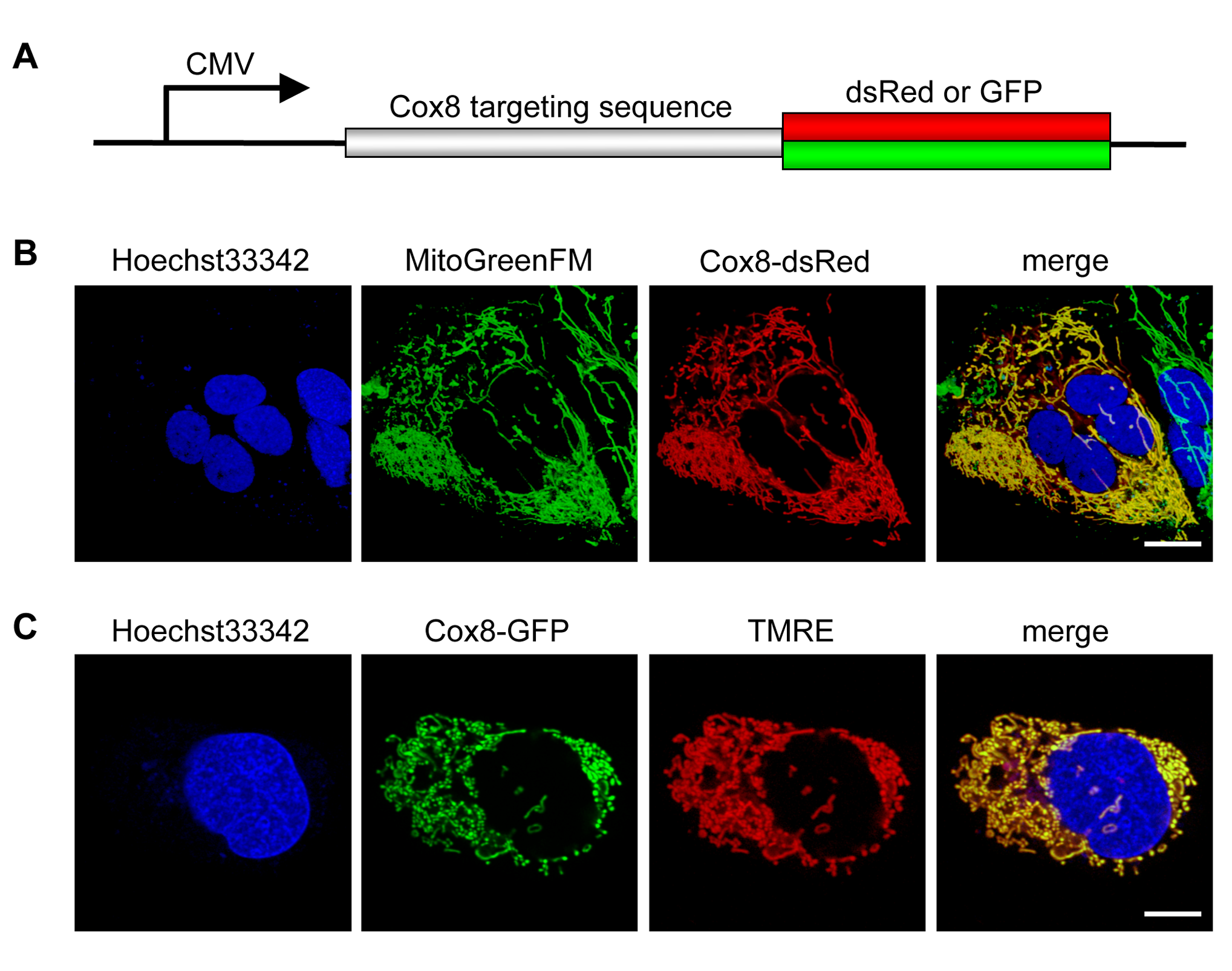

Supplement: Figure S3 — Cox8-GFP and Cox8-dsRed localize to the host cell mitochondria. A DsRed or GFP were fused to the targeting sequence of the mitochondrial protein Cox8. Expression in the host cell was driven by the CMV promoter. B HepG2 cells were transfected with pDsRed1-N1-Cox8 to label host cell mitochondria with dsRed (red). Cells were then stained with MitoTracker GreenFM (green) to confirm localization to mitochondria. Nuclei were labeled blue with Hoechst 33342. Bar = 10 µm, CPS. B HepG2 cells were transfected with pEGFP-N1-Cox8 to label host cell mitochondria with GFP (green). Cells were then stained with TMRE (red) to confirm localization to mitochondria and Hoechst 33342 (blue) to visualize nuclei. Bar = 10 µm, CPS. (TIF) [file ppat.1002224.s003.tif]
